# Determine Cumulative Radiation Dose and Lifetime Cancer Risk in Marfan Syndrome Patients Who Underwent Computed Tomography Angiography of the Aorta in Northeast Thailand: A 5-Year Retrospective Cohort Study

**DOI:** 10.3390/tomography8010010

**Published:** 2022-01-05

**Authors:** Narumol Chaosuwannakit, Phatraporn Aupongkaroon, Pattarapong Makarawate

**Affiliations:** 1 Radiology Department, Faculty of Medicine, Khon Kaen University, Khon Kaen 40000, Thailand; phatraaupong@gmail.com; 2 Cardiology Unit, Internal Medicine Department, Faculty of Medicine, Khon Kaen University, Khon Kaen 40000, Thailand; nchaosuw@gmail.com

**Keywords:** Marfan syndrome, CTA, radiation dose, lifetime cancer risk, CTA aorta

## Abstract

Objective: To evaluate computed tomography angiography (CTA) data focusing on radiation dose parameters in Thais with Marfan syndrome (MFS) and estimate the distribution of cumulative radiation exposure from CTA surveillance and the risk of cancers. Methods: Between 1st January 2015 and 31st December 2020, we retrospectively evaluated the cumulative CTA radiation doses of MFS patients who underwent CTA at Khon Kaen University Hospital, a leading teaching hospital and advanced tertiary care institution in northeastern Thailand. We utilized the Radiation Risk Assessment Tool (RadRAT) established at the National Cancer Institute in Bethesda, Maryland, to evaluate the risk of cancer-related CTA radiation. Results: The study recruited 29 adult MFS patients who had CTA of the aorta during a 5-year study period with 89 CTA studies. The mean cumulative CTDI vol is 21.5 ± 14.68 mGy, mean cumulative DLP is 682.2 ± 466.7 mGy.cm, the mean baseline future risk for all cancer is 26,134 ± 7601 per 100,000, and the excess lifetime risk for all cancer is 2080.3 ± 1330 per 100,000. The excess lifetime risk of radiation-induced cancer associated with the CTA surveillance study is significantly lower than the risk of aortic dissection or rupture and lower than the baseline future cancer risk. Conclusions: We attempted to quantify the radiation-induced cancer risk from CTA surveillance imaging performed for MFS patients in this study, with all patients receiving a low-risk cumulative radiation dose (less than 1 Gy) and all patients having a low excessive lifetime risk of cancer as a result of CTA. The risk–benefit decision must be made at the point of care, and it entails balancing the benefits of surveillance imaging in anticipating rupture and providing practical, safe treatment, therefore avoiding morbidity and mortality.

## 1. Introduction

**Figure 1:**
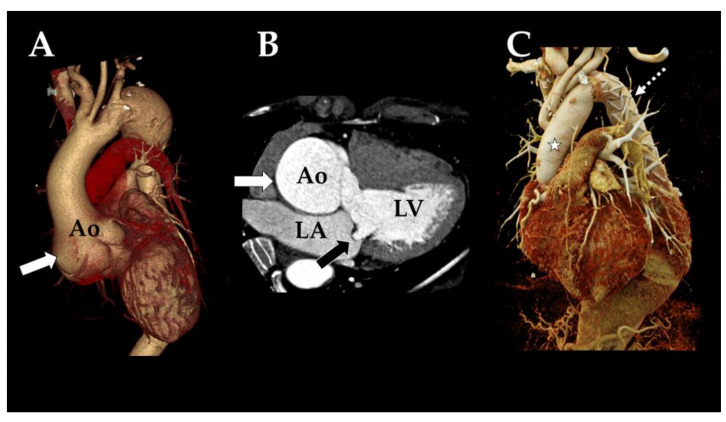
Thoracic aorta CTA findings in Marfan syndrome patients: The 3D volume rendering technique image (**A**) and three-chamber view image (**B**) showed dilated aortic root in tulip bulb configuration and annuloaortic ectasia (white arrow). Mitral valve prolapse is demonstrated (**B**: black arrow). The post-operative CTA image demonstrated the normal size of the vascular graft at ascending thoracic aorta (**C**: star) and evidence of thoracic endovascular aortic repair (**C**: dashed arrow). (Ao; aorta, LA; left atrium, LV; left ventricle).

Marfan syndrome (MFS) is a connective tissue disorder that affects the ocular, musculoskeletal, and cardiovascular systems. The majority of patients would suffer from their aortic root pathology, which would manifest as either a fatal aortic aneurysm rupture or dissection complications [1,2,3]. Since preventive surgery can prevent aortic dissection and rupture, early detection and diagnosis are essential [2,3]. Patients with MFS now receive multidisciplinary management with more frequent surveillance and early preventative surgery, which has resulted in increased lifespan and decreased emergency surgeries. Over the last three decades, prestigious scientific medical centers have pioneered modern MFS care, including preventing and treating life-threatening cardiovascular complications [4,5,6,7]. Aortic enlargement is typically the largest at the sinuses of Valsalva in MFS, which causes annuloaortic ectasia (Figure 1). This pattern can also be noticed in individuals who may not have the Marfan phenotype [1,2,3]. Once aortic dilatation is indicated based on echocardiography, a computed tomography angiography (CTA) or magnetic resonance imaging (MRI) is performed to establish the diagnosis and determine management decisions for aortic aneurysms. This is exceptionally crucial when diameters are on the borderline to determine whether or not to proceed with intervention and monitor enlargement rates during follow-up to ensure that the entire aorta is visualized and the affected regions are identified [6,7,8]. Diagnostic follow-up utilizing MRI or CTA at periodic intervals is recommended for MFS patients treated without surgical or endovascular intervention, according to the European Society of Cardiology (ESC) guidelines on the diagnosis and management of aortic diseases [9]. Since echocardiography does not allow for the visualization of the whole thoracic aorta, a periodic MRI or CTA, as appropriate, is required. On a patient-by-patient basis, however, the rate of development and the risk of dissection is unpredictable [8,9,10]. Because of its availability, rapidity, and excellent spatial resolution, CTAs have been performed and recommended for serial follow-up of MFS patients (Figure 1) [8,9,10,11]. 

Patients with MFS are subjected to repeated CTAs of the aorta, resulting in substantial lifetime radiation doses. Several studies on MFS’s clinical outcomes and CTA data have been published, even though they are primarily from Western populations [8,9,10]. Moreover, no CTA data regarding Thai MFS patients have ever been reported. The purpose of this study was to assess the CTA data with a focus on radiation dose parameters of Thai individuals with Marfan syndrome, and to estimate the distribution of cumulative radiation dose from CTA surveillance and associated lung and all organ cancer risk.

## 2. Materials and Methods

### 2.1. Patient Population

**Figure 2:**
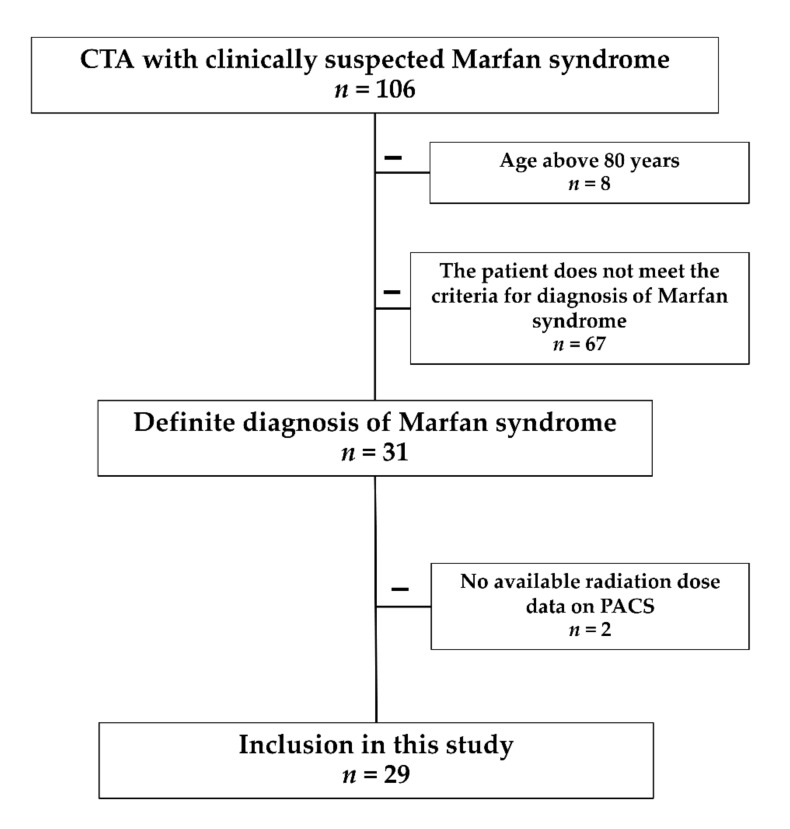
Flowchart for the patients’ inclusion and exclusion in the study.

We retrospectively reviewed the accumulative CT radiation doses of MFS patients who underwent CTA at Khon Kaen University Hospital, the principal teaching hospital and advanced tertiary care institution in northeastern Thailand, between 1st January 2015 and 31st December 2020. Patient identification was made by reviewing our institution’s picture archiving and communication system (PACS) records data. The local Ethics Committee of Khon Kaen University, Thailand, reviewed and approved this study and was registered under reference number HE641421. The study was conducted according to the Declaration of Helsinki principles. All methods were performed in accordance with the relevant guidelines and regulations. The local Ethics Committee of Khon Kaen University also approved our investigation with a waiver of informed consent due to retrospective study design, and patient confidentiality was protected. Cardiologists and ophthalmologists perform a comprehensive physical examination, particularly in the ocular, cardiovascular, and musculoskeletal systems, giving MFS patients a definite diagnosis. The revised Ghent’s nosology for Marfan syndrome has been used to establish the MFS diagnosis [2]. A diagnosis of definite MFS requires at least two systems with major criteria and one additional system involvement, either major or minor. We identified all MFS adult patients (age >15 years) who underwent one or more CT scans, including at least two cancer-sensitive tissue organs or areas (brain, neck, spine, chest, abdomen, and pelvis). The exclusion criteria were the missing radiation dose data in the PACS, >80 years of age, or death during or after the hospital admission (Figure 2).

### 2.2. Computed Tomography Angiography (CTA)

We had three CT scanners during the study period: a 128-slice Optima CT 660 (GE Healthcare, North Richland Hills, TX, USA), a Brilliance iCT scan 128 (Philips Healthcare, Amsterdam, The Netherlands), and a dual-source Somatom definition Flash (Siemens Healthineers, Forchheim, Germany). Standard protocol for CTA aorta was performed on all studies.

### 2.3. Definition and Dosimetry

The computed tomography dose index volume (CTDI_vol_) was provided for a 32 cm CTDI phantom. The CTDI_vol_ is dependent on the imaging acquisition and the exposure parameters input such as the peak kilovoltage (kVp), the product of current and exposure time (mAs), bundle filtering, collimation, and pitch. The CTDI_vol_ and dose length product (DLP) were recorded for each CT examination. The patient dose data, CTDI_vol_, and DLP) values were extracted from the picture archiving and communication system (PACS). A summary of the two relevant dose parameters is given below, along with methods for calculating these factors. The Dose Length Product (DLP, units: mGy.cm) indicates the mean absorbed dose to the patient of each sequence in a CT exam and is calculated by multiplying CTDI_vol_ by the scan length. It measures the total CT examination’s mean effective dose to the patient [12,13]. The CT monitor’s real-time CTDI_vol_ and DLP displays were collected in the PACS and retrospectively analyzed.

### 2.4. Estimated Lifetime Cancer Risk

The Radiation Risk Assessment Tool (RadRAT), developed at the National Cancer Institute in Bethesda, MD, and available at https://radiationcalculators.cancer.gov/radrat/ (accessed on 1 October 2021), was used to estimate the risk of cancer-associated by CTA radiation [14]. The Radiation Risk Assessment tool (RadRAT) was developed primarily utilizing data from survivors of the Hiroshima and Nagasaki atomic explosions and patients receiving radiation from the Biological Effects of Ionizing Radiation (BEIR) VII report [14]. The following information is necessary to estimate the cancer risk, as indicated on the RadRAT workflow: Patient’s sex and year of birth, number of exposures, exposure event, year of each exposure, exposed regions (organ), exposure rate (chronic or acute), and exposure dose distribution with related characteristics are all part of the exposure history. The lifetime risk estimates calculator was created using the Analytica programming software and Monte Carlo simulation methods using Latin hypercube sampling to predict a distribution of potential lifetime risk estimates. Gonzalez et al. described the methodology, and statistical calculations used to estimate the risk of radiation-induced cancer [14]. 

### 2.5. Statistical Analysis

Continuous data were expressed as mean ± SD. A significance level of *p* < 0.05 was considered a statistically significant result, and all reported *p*-values were two-sided. Means were compared using unpaired *t*-test, and Mann–Whitney rank sum was used when data were not normally distributed. All the patient’s demographics are based on their initial scan. According to Kolmogorov–Smirnov tests, the distributions of cumulative CTDI_vol_, cumulative DLP, and the excess lifetime risk for cancer were not substantially different from normal. Hence, they are provided as mean and SD. The correlation between different variables was determined using Pearson’s correlation equation. The difference between the two data sets was assessed using repeated analysis of variance (ANOVA) with Greenhouse–Geisser. The Rank-sum test compared different parameters, lifetime attributable risk (LAR) cancer incidence, and cumulative effective doses between emergency surgery and elective surgery patients. The lifetime risk of developing cancer of the ionizing radiation (chances in 100,000) between baseline future risk and excessive lifetime risk to the end of the expected lifetime were compared using Fisher’s exact test.

## 3. Results

**Figure 3:**
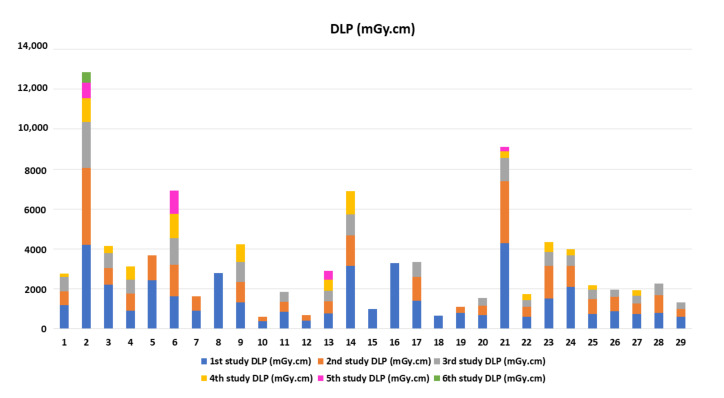
The cumulative dose length product (DLP) of each individual.

**Figure 4:**
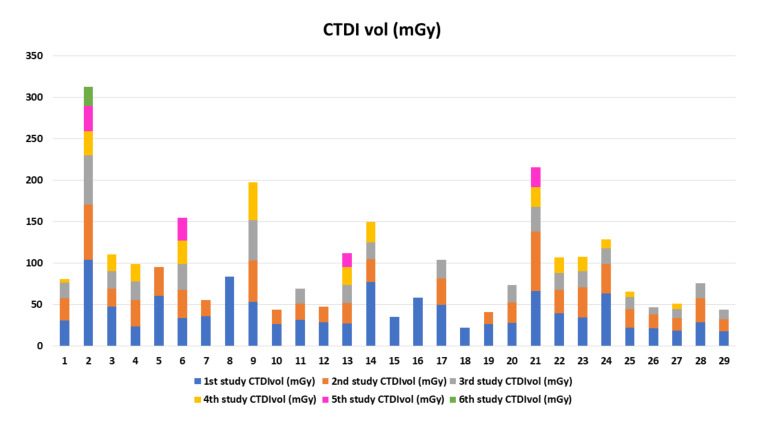
The cumulative computed tomography dose index volume (CTDI_vol_) for each individual.

**Figure 5:**
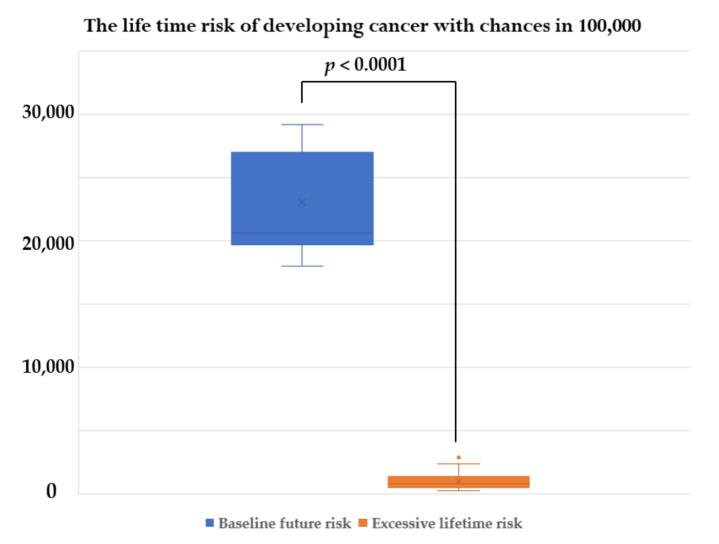
Comparison between the lifetime risk of developing cancer with chances in 100,000 between baseline future risk and the excessive lifetime risk of developing cancer.

The study recruited 29 adult MFS patients who had CTA of the aorta during a 5-year study period with 89 CTA studies. The demographic data, clinical features, and outcomes of MFS patients are demonstrated in Table 1. The mean age at diagnosis is 31.1 years. The operative and radiation dose information is shown in Table 2. There is no statistically significant between the patients who underwent emergency surgery and elective surgery of age, gender, weight, height, body mass index (BMI), and underlying hypertension. The most frequent cause for emergency surgery is aortic dissection with Stanford type A (*n* = 6), followed by complicated aortic dissection, Stanford type B (*n* = 2), and aortic rupture (*n* = 1). The emergency surgery group underwent repeat operation and CTA more frequently than the elective surgery group (*p* = 0.01 and *p* = 0.04, respectively). The cumulative CT radiation exposure of each patient was determined, and the biological effects of the ionizing radiation approach were used to estimate the lifetime risk of cancer. The emergency surgery group had significantly higher cumulative CTDI_vol_, DLP, excessive lifetime risk for all cancer, and excessive lifetime risk for lung cancer. The baseline future risk for all cancers is not statistically significant between the emergency surgery and elective surgery groups (Table 2). Summary data for CTA counts, radiation dose, the excess lifetime risk of all cancer, excessive lifetime risk for lung cancer, the baseline future risk for all cancer, and the total future risk (per 100,000) are shown in Table 3. The cumulative DLP and CTDI_vol_ for each individual are shown in Figure 3 and Figure 4, respectively. The mean cumulative CTDI_vol_ is 21.5 ± 14.68 mGy, mean cumulative DLP is 682.2 ± 466.7 mGy.cm, the mean baseline future risk for all cancer is 26,134 ± 7601 per 100,000, and the excessive lifetime risk for all cancer is 2080.3 ± 1330 per 100,000 radiation doses. The excessive lifetime risk of radiation-induced cancer associated with the CTA surveillance study is significantly lower than the baseline future risk of cancer and lower than the risk of aortic dissection or rupture (*p* < 0.0001) (Figure 5). 

## 4. Discussion

Computed tomography angiography (CTA) is more generally available, less expensive, and maybe performed safely in patients with pacemakers and other MRI contraindications. Multidetector CTA allows for faster scanning of the aorta with isotropic voxel data and higher spatial resolution. Aneurysm detection sensitivity, specificity, and accuracy have all increased considerably with modern-generation CT scanners [8,15,16]. To our knowledge, our report represents the first analysis of MFS patient radiation dose who underwent CTA in Thailand. It is hoped that this will stimulate interest in the region to benefit patients and staff and create awareness of radiation safety among clinicians and radiologists. In the present study, we demonstrated cumulative radiation dose, and the radiation risk calculator estimates lifetime attributable risk from the time of exposure until the end of the expected lifetime in Marfan syndrome patients who underwent CTA.

The present study demonstrated that the lifetime risk of radiation-induced all cancers (2080.3 ± 1330.1 per 100,000) and lung cancer (288.4 ± 214.8 per 100,000) associated with the CTA surveillance study is significantly lower than the risk of aortic dissection or rupture (1.33% of MFS with an aortic diameter of 50 to 54 mm had aortic adverse events and aortic diameter ≥50 mm, the danger rose fourfold) [17,18,19,20,21]. Malhotra A. et al. discovered that the primary factors contributing to an excess lifetime risk are obtaining CTA follow-up at a younger age, more frequent follow-up, a longer surveillance duration, and male gender [22]. This is comparable to the emergency surgery group in the present study, which had significantly higher cumulative CTDIvol, DLP, excess lifetime risk for all cancer, and excessive lifetime risk for lung cancer. 

The National Cancer Institute established the RadRAT tool for assessing radiation-linked lifetime risk given a particular exposure history [14]. The risk is determined by sex, achieving age, and exposure age. It summarizes the risk associated with each exposure as well as the overall risk associated with all exposures. The program evaluates the cumulative excessive lifetime risk following exposure since the risk of radiation-related cancer remains increased for at least 50 years. The risk–benefit decision between the cancer risk versus the advantages of surveillance imaging to prevent complications must be taken at the patient level. It requires considering the advantages of surveillance imaging in predicting rupture and providing effective, safe treatment, hence reducing morbidity and mortality.

Since the chance of mortality from developing more than one radiation-related cancer because of a single exposure is extremely low for doses below 1 Gy, the lifetime attributable risk and risk of exposure-induced cancer (REIC) are almost similar for the radiation doses per scan evaluated in RadRAT [14,17,18,19,20]. The increased use of CTA raises radiation concerns since cumulative CT radiation exposure from several CTA examinations adds progressively to baseline cancer risk. Observational studies to determine CTA-related cancer risks would be impossible to conduct since they would require large studies and long-term follow-up. The increased risk of benign and malignant tumors of the lung and other organs is likely insignificant compared to the chance of aortic dissection or rupture in Marfan syndrome patients, which can cause considerable morbidity and death [6,7,13,14,15,16]. The risk of radiation-related cancer has been demonstrated to remain elevated for at least fifty years after exposure to radiation, effectively for the remainder of a person’s life. As a result, the cumulative excess lifetime risk, computed as the sum of the age-specific hazards adjusted for the chance of surviving to that age, is a typical summary statistic for reflecting the whole potential detriment of an exposure. The radiation risk calculator calculates lifetime attributable risk from the period of the exposure until the end of the predicted lifetime [12,13,14]. When assessing the risk of radiation-induced cancer, the variation in radiation doses from each exposure must be taken into account. Patient size, imaging parameters (scan length, tube voltage, and tube current-time product), and scanner technology affect CT effective dose [8,23]. Effective dosages of 1.1–9.4 mSv have been reported for CTA in assessing Marfan syndrome patients [8,23,24]. Radiation doses associated with CTA are reducing incrementally because of advanced technology like dual-source CTA and strategies including iterative reconstruction [8,23,24,25]. According to the International Commission on Radiological Protection, absorbed doses up to 100 mGy do not cause deterministic radiation effects or clinically relevant functional impairment in any tissue [17,20]. Because of the delayed effect of radiation and the extremely low incidence of radiation-induced cancer, determining such risks directly through observational studies would generally need unfeasibly large studies with long-term follow-up to achieve relevant statistical power. 

Even though there are promising results, there are some limitations and considerations to keep in mind. Firstly, our research was limited to the general Thai population, with an emphasis on Asians. Certain groups and individuals may be more radiosensitive and risk developing cancer after ionizing radiation. Secondly, since we did not evaluate CTA performed at other hospitals before the patient was referred to our hospital, we possibly underestimated the cumulative effective doses. Furthermore, interventional radiology, nuclear medicine, invasive angiography, and other radiography studies were excluded from the study. Thirdly, due to retrospective study design, we cannot have pharmacological treatment information and the impact of the confounding factors in the recruited patients. Finally, the present study was conducted in the single-centered with a small sample size due to the rarity of the disease in our population. Future prospective study design, the multi-centered, and larger sample size should be performed to address the importance of the present study results.

## 5. Conclusions

We attempted to quantify the risk of radiation-induced cancer from CTA surveillance imaging performed for MFS patients in this study, with all patients receiving a low-risk cumulative radiation dose (less than 1 Gy). The excess lifetime risk of radiation-induced cancer associated with the CTA surveillance study (0.02%) is significantly lower than the baseline future risk of cancer (26%) and lower than the risk of aortic dissection or rupture. (1.33% of MFS with an aortic diameter of 50 to 54 mm had aortic adverse events, and with aortic diameter ≥50 mm, the danger rose fourfold). The risk–benefit decision must be taken at the point of care, and it requires balancing the benefits of surveillance imaging in predicting rupture against the benefits of practical, safe treatment, therefore minimizing morbidity and mortality.

## Figures and Tables

**Table 1:** Patient demographics, clinical features, and outcomes of patients with Marfan syndrome.

Features	
Age at diagnosis (years), mean ± SD	31.1 ± 9.4
Male, *n* (%)	13 (44.8)
Weight (kg), mean ± SD	60.9 ± 10.2
Height (cm), mean ± SD	171.9 ± 6.8
BMI (kg/m2), mean ± SD	20.4 ± 2.3
Family history of Marfan syndrome	10 (34.5)
No surgery, *n* (%)	3 (10.3)
Number of surgical interventions, mean ± SD	1.8 ± 1.1
Post-operative follow-up (years), mean ± SD	5.1 ± 2.2
HT, *n* (%)	5 (17.2)
Smoking, *n* (%)	2 (6.9)

SD, standard deviation; BMI, body mass index; HT, hypertension.

**Table 2:** Patient demographic data, operative, and radiation dose information, the excessive lifetime risk of all cancer, excessive lifetime risk for lung cancer, the baseline future risk for all cancer, and the total future risk (per 100,000).

Feature	Emergency Surgery *n* = 9	Elective Surgery *n* = 20	*p*-Value (95% CI)
Age (years), mean ± SD	35.8 ± 7.3	29.1 ± 9.7	0.07 (−14.16 to 0.76)
Male, *n* (%)	4 (44.4)	9 (45)	0.97 (−34.09 to 33.54)
Weight (kg), mean ± SD	62.4 ± 10.0	60.3 ± 10.5	0.62 (−10.63 to 6.42)
Height (cm), mean ± SD	172.1 ± 5.9	171.8 ± 7.3	0.91 (−5.99 to 5.39)
BMI (kg/m2), mean ± SD	20.9 ± 2.1	20.1 ± 2.3	0.38 (−2.64 to 1.05)
Aortic dissection Stanford type A, *n* (%)	6 (66.7)	0 (0)	0.0001 (31.5 to 87.96)
Aortic dissection, Complicated Stanford type B, *n* (%)	2 (22.2)	2 (10)	0.38 (−13.4 to 45.51)
Aortic rupture, *n* (%)	1 (11.1)	0 (0)	0.13 (−7.4 to 43.5)
Rapid growth (>10 mm/y), n (%)	0 (0)	2 (10)	0.33 (−20.8 to 30.1)
Family history of Marfan syndrome, *n* (%)	1 (11.1)	2 (10)	0.93 (−20.9 to 34.3)
Repeat operation, *n* (%)	4 (44.4)	1 (5)	0.01 (7.8 to 68.6)
Number of operations, mean ± SD	2.2 ± 1.1	1.2 ± 1.2	0.07 (−1.9 to 0.06)
HT, *n* (%)	2 (22.2)	3 (15)	0.64 (−19.2 to 41.2)
Smoking, *n* (%)	1 (11.1)	1 (5)	0.55 (−14.6 to 38.7)
Cumulative CTA count, mean (range)	3.8, (3–6)	2.8, (1–5)	0.04 (−1.9 to −0.04)
Cumulative CTDIvol (mGy), mean ± SD	44.8 ± 17.53	26.7 ± 12.86	0.0042 (−29.9 to −6.2)
Cumulative DLP (mGy.cm), mean ± SD	1100.78 ± 686.1	712.9 ± 316.3	0.04 (−765.2 to −10.6)
The excessive lifetime risk for all cancer *	2005.3 ± 330.1	1713.5 ± 226.0	0.009 (−506.9 to −76.7)
The excessive lifetime risk for lung cancer	352.9 ± 24.8	259.3 ± 35.8	<0.0001 (−120.7 to −66.5)
The baseline future risk for all cancer **	25943.9 ± 6601.4	26219.7 ± 6257.2	0.91 (−4963.1 to 5514.7)

BMI, body mass index; CTA, computed tomography angiography; DLP, dose length product; CTDI_vol_, computed tomography dose index volume; SD, standard deviation. * The lifetime risk of developing cancer of the ionizing radiation (chances in 100,000) with a 90% uncertainty range and risk from the time of exposure to the end of the expected lifetime. ** Risk from 2021 to the end of the expected lifetime *p*-value < 0.05 is considered statistically significant.

**Table 3:** Summary data for CTA counts, radiation dose, the excessive lifetime risk of all cancer, excessive lifetime risk for lung cancer, the baseline future risk for all cancer, and the total future risk (per 100,000).

Cumulative CTA Count, Mean (Range)	1 (1–6)
Cumulative CTDI vol (mGy), mean ± SD	21.5 ± 14.68
Cumulative DLP (mGy * cm), mean ± SD	682.2 ± 466.7
The excessive lifetime risk for all cancer *	2080.3 ± 1330.1
The excessive lifetime risk for lung cancer	288.4 ± 214.8
The baseline future risk for all cancer **	26,134.1 ± 7601.4
The total future risk for all cancer **	27,509.3 ± 9208.2

CTA, computed tomography angiography; DLP, dose length product; CTDIvol, computed tomography dose index volume; SD, standard deviation. * The lifetime risk of developing cancer of the ionizing radiation (chances in 100,000) with a 90% uncertainty range and risk from the time of exposure to the end of the expected lifetime. ** Risk from 2021 to the end of the expected lifetime.

## Data Availability

Not applicable.
